# Using Mobile Apps to Assess and Treat Depression in Hispanic and Latino Populations: Fully Remote Randomized Clinical Trial

**DOI:** 10.2196/10130

**Published:** 2018-08-09

**Authors:** Abhishek Pratap, Brenna N Renn, Joshua Volponi, Sean D Mooney, Adam Gazzaley, Patricia A Arean, Joaquin A Anguera

**Affiliations:** ^1^ Department of Biomedical Informatics and Medical Education School of Medicine University of Washington Seattle, WA United States; ^2^ Sage Bionetworks Seattle, WA United States; ^3^ Department of Psychiatry & Behavioral Sciences University of Washington Seattle, WA United States; ^4^ Department of Neurology University of California San Francisco San Francisco, WA United States; ^5^ Department of Psychiatry University of California San Francisco San Francisco, CA United States

**Keywords:** mobile apps, smartphone, depression, Hispanics, Latinos, clinical trial, cognition, problem solving, mHealth, minority groups

## Abstract

**Background:**

Most people with mental health disorders fail to receive timely access to adequate care. US Hispanic/Latino individuals are particularly underrepresented in mental health care and are historically a very difficult population to recruit into clinical trials; however, they have increasing access to mobile technology, with over 75% owning a smartphone. This technology has the potential to overcome known barriers to accessing and utilizing traditional assessment and treatment approaches.

**Objective:**

This study aimed to compare recruitment and engagement in a fully remote trial of individuals with depression who either self-identify as Hispanic/Latino or not. A secondary aim was to assess treatment outcomes in these individuals using three different self-guided mobile apps: iPST (based on evidence-based therapeutic principles from problem-solving therapy, PST), Project Evolution (EVO; a cognitive training app based on cognitive neuroscience principles), and health tips (a health information app that served as an information control).

**Methods:**

We recruited Spanish and English speaking participants through social media platforms, internet-based advertisements, and traditional fliers in select locations in each state across the United States. Assessment and self-guided treatment was conducted on each participant's smartphone or tablet. We enrolled 389 Hispanic/Latino and 637 non-Hispanic/Latino adults with mild to moderate depression as determined by Patient Health Questionnaire-9 (PHQ-9) score≥5 or related functional impairment. Participants were first asked about their preferences among the three apps and then randomized to their top two choices. Outcomes were depressive symptom severity (measured using PHQ-9) and functional impairment (assessed with Sheehan Disability Scale), collected over 3 months. Engagement in the study was assessed based on the number of times participants completed active surveys.

**Results:**

We screened 4502 participants and enrolled 1040 participants from throughout the United States over 6 months, yielding a sample of 348 active users. Long-term engagement surfaced as a key issue among Hispanic/Latino participants, who dropped from the study 2 weeks earlier than their non-Hispanic/Latino counterparts (*P*<.02). No significant differences were observed for treatment outcomes between those identifying as Hispanic/Latino or not. Although depressive symptoms improved (beta=–2.66, *P*=.006) over the treatment course, outcomes did not vary by treatment app.

**Conclusions:**

Fully remote mobile-based studies can attract a diverse participant pool including people from traditionally underserved communities in mental health care and research (here, Hispanic/Latino individuals). However, keeping participants engaged in this type of “low-touch” research study remains challenging. Hispanic/Latino populations may be less willing to use mobile apps for assessing and managing depression. Future research endeavors should use a user-centered design to determine the role of mobile apps in the assessment and treatment of depression for this population, app features they would be interested in using, and strategies for long-term engagement.

**Trial Registration:**

Clinicaltrials.gov NCT01808976; https://clinicaltrials.gov/ct2/show/NCT01808976 (Archived by WebCite at http://www.webcitation.org/70xI3ILkz)

## Introduction

Technology is being leveraged as a way to perform large-scale clinical research targeting typically underrepresented populations. Given the extensive use of mobile devices across communities, remote research methods are becoming widely used. Additionally, technology is also seen as a potential method for bridging health disparities, which are typically driven by limited resources and stigma most apparent in minority communities. Of particular interest is the Hispanic/Latino community: Although they comprise one of the fastest-growing demographic segments in the United States [1], Hispanic/Latino populations are half as likely as their non-Hispanic white counterparts to receive mental health services [2]. This population is very difficult to recruit into research [3,4], and as a result, there is limited science to support treatment recommendations for this population. Recruitment of Hispanic/Latino samples into clinical research is particularly challenging in studies of mental health.

The widespread availability of digital technology has the potential to drive a sea change in access to psychosocial treatment for mental health problems in Hispanic/Latino communities [5]. Internet-based interventions have already demonstrated comparable treatment outcomes as traditional face-to-face psychotherapy [6], and given that 75% of Hispanic/Latino individuals own a smartphone [1], mobile-based mental health apps have the potential to increase treatment accessibility and engagement. Although there is potential for treating depression in Hispanic/Latino individuals using mobile devices, there is relatively little information about how this population interacts with apps, given their underrepresentation in mental health research. In particular, whether Hispanic/Latino smartphone owners (including both Spanish and English speakers) actually use mental health apps, and when they do, whether they follow the app protocols. We recently tested similar questions among a majority non-Hispanic white sample in a recent, fully remote trial (BRIGHTEN V1 [7,8]) and found that their interest in depression apps was high. It was far less challenging to recruit participants into our remote clinical trial compared with traditional in-person treatment trials. However, long-term engagement with the assigned apps trailed off significantly each week in the study, a finding that has been demonstrated in other studies [9]. However, Hispanic/Latino individuals, especially non-English speakers, do not typically have the same opportunity as majority groups to utilize mental health services and therefore may find mental health apps a useful alternative to traditional care. There is an immediate need for further research to develop and evaluate new solutions for mental health care for this population that are economically viable, scalable, and focused on engaging users to inform timely and evidence-based clinical interventions.

Therefore, the aim of this study was to determine the feasibility of conducting remote research with a Hispanic/Latino adult sample of smartphone users, how they interact with depression apps, and the potential clinical impact mHealth apps may have on treating depression in this population. We report recruitment, engagement, and cost in this 12-week, fully remote randomized controlled trial among Hispanic/Latino individuals with depression and a cohort of non-Hispanic/Latinos with depression to act as a direct comparator group (and extend our previous findings).

## Methods

### Approval

Ethical approval for the trial (NCT01808976) was granted by the Institutional Review Board of University of California, San Francisco. Specific research methods for this project replicated the BRIGHTEN V1 study and are described elsewhere [7,8], but are summarized here. Briefly, this was a fully remote treatment trial for depression, consisting of engagement with one of three treatment apps and periodic assessments detailed below.

### Recruitment

**Figure 1:**
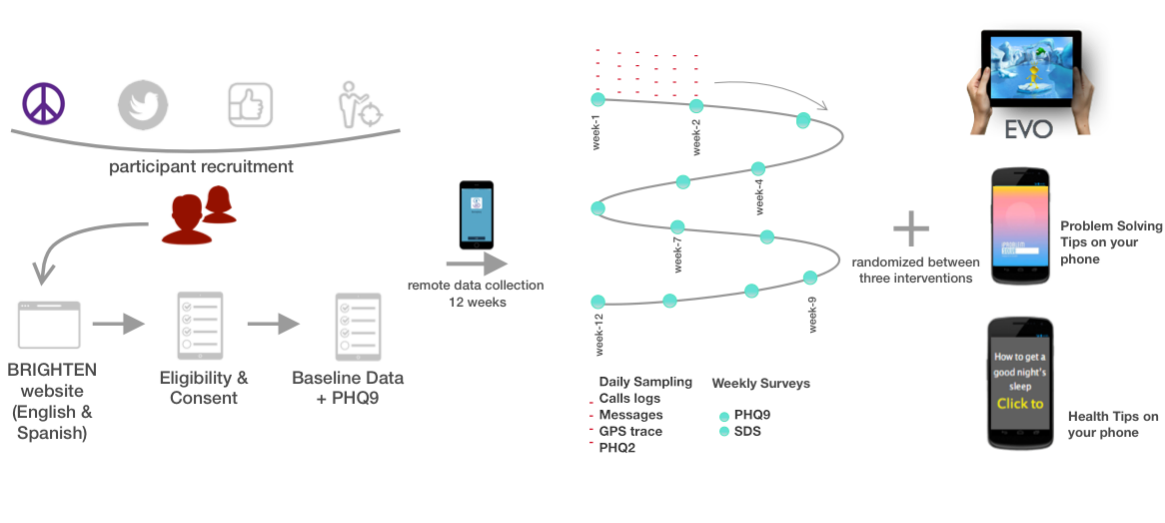
Overall BRIGHTEN V2 study schematic showing participant recruitment, consent, enrollment, and randomization workflow along with weekly and daily data collection. EVO: Project Evolution; GPS: Global Positioning System; PHQ-2: 2-item Patient Health Questionnaire; PHQ-9: 9-item Patient Health Questionnaire; SDS: Sheehan Disability Scale.

Three different types of recruitment approaches, including traditional, social networking, and search-engine strategies, were used (Figure 1). Traditional methods consisted of Craigslist.org postings throughout the United States, specifically posting to the “Volunteer” and “Jobs etc” pages within Craigslist in at least one major city in every state. Social networking methods included regular postings on sites such as Facebook and Twitter and contextual-targeting methods to identify and directly push recruitment ads to potential participants, based on their Twitter and other social media comments. This approach was led entirely by Trialspark.com, which designed specific recruiting campaigns using machine learning approaches to create optimal advertising. Furthermore, we reached out to Hispanic/Latino Catholic Ministries in at least one city in every state to see if they would be willing to help with the recruitment for this study and post fliers in their communities. Each approach (described further in Multimedia Appendix 1 provided potentially interested participants a link to our custom study website, which was translated entirely for Spanish speakers and included a welcome video featuring bilingual Hispanic/Latino researchers describing the goal of this study in Spanish. All translations involving text in the treatment apps were done by a combination of native Spanish speakers associated with this study and professionals at Babble-on.

### Procedures

This study used an equipoise stratified clinical trial design [10], which factors participant preferences for treatment into randomization. Participants were randomly assigned one app among their two preferred intervention types and were asked to use it daily for 4 weeks. Participants completed primary outcome assessments, including the Patient Health Questionnaire-9 (PHQ-9) [11] and Sheehan Disability Scale (SDS) [12] once a week for 3 months, with other secondary measures (described below) completed at daily, weekly, or biweekly intervals. All treatments and assessments were delivered remotely via custom apps.

#### Screening

Interested participants completed a brief Web-based screening consisting of questions about their ability to speak Spanish (“Do you speak Spanish?; ¿Hablas Español?”) and mobile device ownership (“Do you have an iPhone or Android smartphone?”).

#### Consent

Participants were given the University of California, San Francisco consent form to read and were instructed to watch a video that highlighted the goals and procedures of the study, as well as risks and benefits of participation. After viewing the video, participants had to pass a quiz that confirmed their understanding that participation was voluntary, was not a substitute for treatment, and that they were to be randomized to treatment conditions. Each question had to be answered correctly before moving on to baseline assessment and randomization. Eligibility was established after consent was obtained. Upon being eligible, participants were sent a link to download their assessment app (Surveytory).

#### Participant Eligibility

Participants had to speak English or Spanish, be 18 years old or older, and own either an iPhone with Wi-Fi or 3G/4G/LTE capabilities or an Android phone along with an Apple iPad version 2.0 or newer device. An iOS-based device was required as one of our intervention apps was only available on iOS devices at the time of the study. If a user had an Android phone, he or she was only eligible to participate if he or she also owned an Apple iPad version 2 or newer iOS tablet device. Participants had to endorse clinically significant symptoms of depression, as indicated by either a score of 5 or higher on PHQ-9 or a score of 2 or greater on PHQ item 10 (indicating feeling disabled in his or her life because of mood).

#### Assessment

##### Baseline

The baseline assessment included collecting demographic variables including age, race/ethnicity, marital and employment status, income, education, smartphone ownership, use of other health apps, and use of mental health services, including use of medications and psychotherapy. We collected information on mental health status using PHQ-9 [11] for depression and SDS [12] to assess self-reported disability. PHQ-9 rates the presence and severity of depressive symptoms across 9 items, with higher scores signifying more severe symptomatology (range 0-27). This is a reliable and well-validated screening instrument [13] that is responsive to depression treatment outcomes over time [11] and is included in the US Preventive Services Task Force recommendations for depression screening in adults [14]. PHQ-9 has been translated into several languages; we used both the original English language form and the validated Spanish translation [15]. The baseline PHQ-9 demonstrated good internal consistency in our sample (Cronbach alpha=.85, 95% CI 0.83-0.87).

SDS assesses perceived functional impairment across 3 domains (work/school, social life, and family/home responsibilities), yielding a sum score of 0-30, in which higher scores represent greater disability. SDS is popular in clinical trials given its sensitivity in detecting treatment effects [16]. As one of the official World Health Organization’s measures of disability, this measure has also been translated into several languages; we used both the original English version and a validated Spanish translation of this scale [17]. SDS also demonstrated good internal consistency in our sample (Cronbach alpha=.89, 95% CI 0.87-0.91).

##### Follow-Up Assessments

Our custom mobile app, Surveytory, was used to collect all outcome and passive data. The assessments to measure changes in mood (PHQ-9) and disability (SDS) were administered weekly. Daily changes in mood were assessed using the PHQ-2 survey. Passive data collection included daily phone usage logs (call/text time, call duration, and text length) and mobility data (activity type and distance traveled using the phone’s accelerometer and Global Positioning System). Participants were automatically notified every 8 hours for 24 hours if they had not completed a survey within 8 hours of its original delivery. A built-in reminder also prompted the participant to check for any surveys on a daily basis in case they missed a new survey notification. An assessment was considered missing if it was not completed within a 24-hour time frame.

##### Treatment

After confirming completion of baseline assessments (or 72 hours after the initiation of these assessments, whichever came first), participants were sent a Web-based survey that described each of the 3 treatment arms. Following this description, participants were asked to select which 2 apps they were most inclined to use in this study. Participants were then randomly assigned to one of these 2 preferred conditions and sent a link to download the intervention app, which included a brief video explaining how to download and use the assigned treatment app. This download also included a custom dashboard to monitor their study progress. Participants were asked to use their assigned app for 1 month.

The first app was a video game-inspired cognitive intervention (Project Evolution, EVO) designed to modulate cognitive control abilities, as declines in these abilities have been associated with depression [18]*.* This intervention has preliminary evidence for being an effective treatment for depression [18]. The second intervention was an app based on internet-based problem-solving therapy (iPST), an evidence-based treatment for depression, which has been shown to be both acceptable and efficacious for US-dwelling Hispanic/Latino populations. The final intervention app, an information control, provided daily health tips (HTips) for overcoming depressed mood such as self-care (eg, taking a shower) or physical activity (eg, taking a walk; see [8] for further descriptions of each).

Each of the 3 apps represented the most common type of self-guided depression apps available at the time of the study: apps based on psychotherapy principles, apps that claim to improve mood through therapeutic games, and apps that provide suggestions for mindfulness and behavioral exercises. Similar to the assessment notifications, each intervention app was equipped with built-in reminders asking the participant to use their app on a daily basis (reminders were sent once daily).

##### Incentives

Randomized participants were paid a total of US $75 in Amazon gift vouchers for completing all assessments over the 12 weeks. Participants received US $15 for completing the initial baseline assessment and an additional US $20 for each subsequent assessment at the 4-, 8-, and 12-week time points.

##### Procedures to Reduce Gaming

“Gaming” is a situation where a user enrolls in a study solely to acquire research payment or attempts to influence specific methodological aspects of the study. We utilized the following safeguards to prevent this: (1) locking the eligibility or treatment randomization survey if a participant tried to change a submitted answer so that only the initial answer was utilized, (2) using study links that are valid for one user/device, and (3) tracking internet protocol addresses to minimize duplicate enrollments.

#### Statistical Analyses

Participant self-reported race/ethnicity was used to create 2 groups of Hispanic/Latino and non-Hispanic/Latino adults (eg, all other races and ethnicities) to test our main study aims. Sample demographics and clinical characteristics were calculated using appropriate descriptive statistics. Comparisons between participant demographics were done using a chi-square test of independence for categorical variables and one-way analysis of variance to compare continuous variables across the groups. To assess the marginal effect (ie, association in the entire sample) between longitudinal weekly PHQ-9 and SDS scores and treatment arms, we used generalized estimating equations (GEEs) [19]. Briefly, GEE models extend generalized linear models to longitudinal or clustered data. GEEs use a working correlation structure that accounts for within-subject correlations of participant responses, thereby estimating robust and unbiased SEs compared with ordinary least squares regression [19,20]. We adjusted for age and gender to account for any potential confounding effects between outcome and main covariates of interest. Treatment response was further categorized into 3 groups based on a change of at least 5 points on PHQ-9 [11], the minimal clinically important difference [11], to comprise treatment responders (decrease PHQ-9≥5 points), nonresponders (change in PHQ-9<5 points), and those that deteriorated over treatment (increase in PHQ-9≥5 points). To assess participant engagement, we examined the proportion of participants who completed at least one activity in any given week. One-way analysis of variance was used to compare the daily, weekly, and overall participation differences between Hispanic/Latino and other participants. Univariate estimation of time to drop out from the study between Hispanic/Latino and non-Hispanic/Latino participants was computed using survival analysis. The distribution of survival days (total days active in the study) and nonparametric estimates of the survivor function was computed using the Kaplan-Meier method [21]. Log-rank test [22] was used to test for differences in survival between Hispanic/Latino and other participants. To compare dropout rates among the 3 interventions, a nonparametric Kruskal-Wallis test was used. Passive data was only used to compare user engagement with active survey-based tasks. Given this study design is similar to that of our previous work [7], we used the same power analysis for this study. It indicated that 200 participants per intervention arm would provide 0.80 power to detect a medium treatment effect (eg, 2 points change on PHQ-9 scale, Cohen *d*=0.4) with an assumption of 50% participant dropout. However, this study was a feasibility trial of an understudied Hispanic/Latino population and was not sufficiently powered to detect a moderate effect size across the 3 interventions. All analyses were carried out using R (R Core Team, Vienna, Austria), statistical computing language version 3.4.2 [23].

## Results

### Recruitment and Enrollment

The BRIGHTEN V2 study started recruitment in August 2016 with screening and enrollment continuing for 7 months. A total of 4502 people were screened, and 23.10% (1040/4502) adults met the eligibility criteria and were enrolled in the study. Of these, 37.40% (389/1040) reported being Hispanic/Latinos. As in BRIGHTEN V1 study [7,8], the use of Craigslist.org was the most effective approach in recruiting, with more than 80% (843/1040) of our participants coming from this approach. An additional 8% (86/1040) were referred by friends or colleagues.

**Figure 2:**
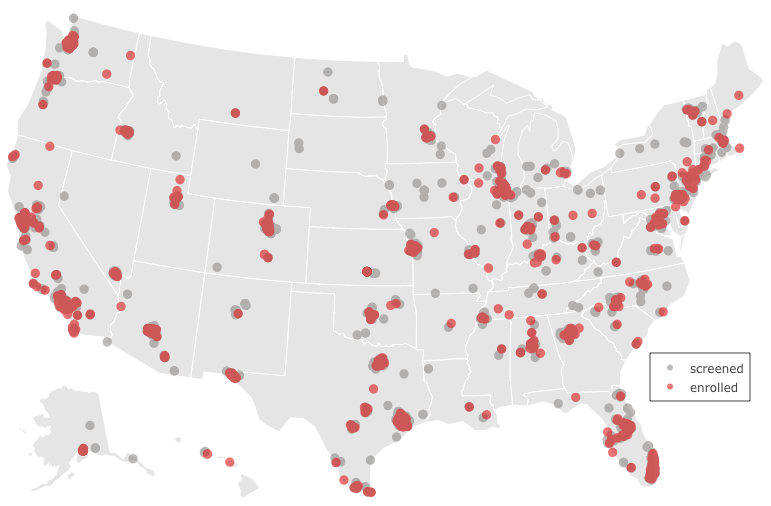
US map showing the location of people who were screened (gray) and enrolled (red) in the BRIGHTEN V2 Study.

**Figure 3:**
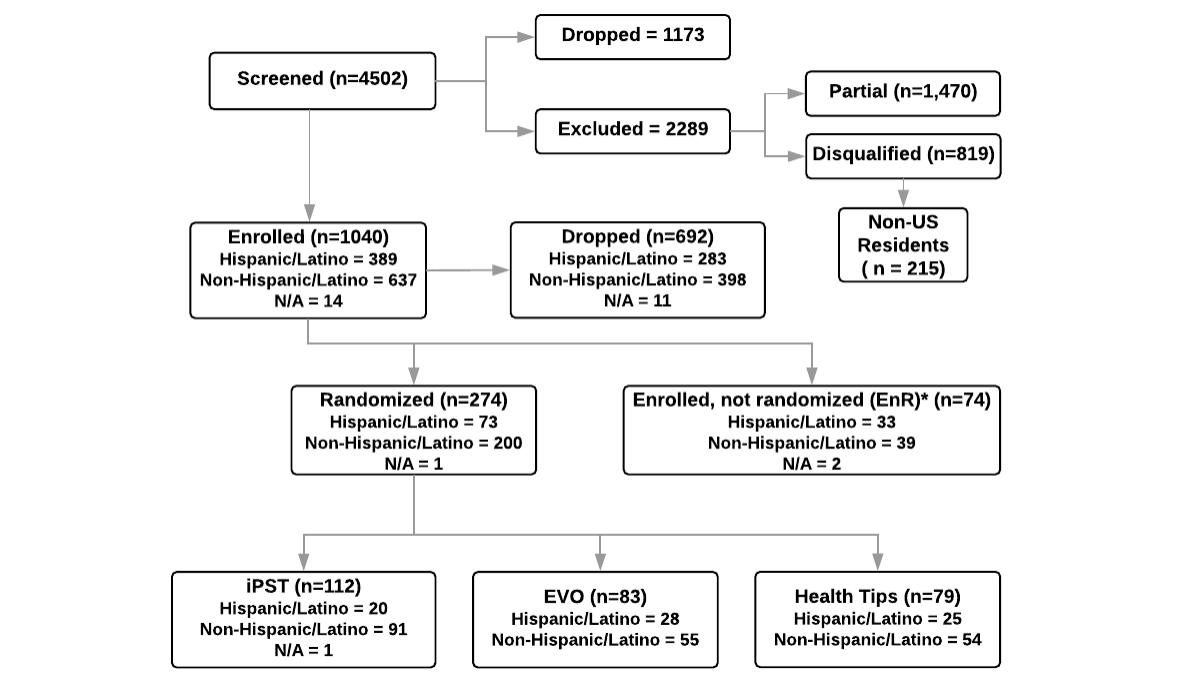
The Consolidated Standards of Reporting Trials flow diagram. iPST: internet-based problem-solving therapy; EVO: Project Evolution; N/A: not available.

Enrolled participants lived throughout the United States, with all the metropolitan areas represented (Figure 2). Only 33.46% (348/1040) of the initially enrolled participants were active in the study (active cohort), as defined by completing at least one postenrollment weekly PHO-9 assessments or providing passive phone usage data within the first 12 weeks. The remaining 66.54% (692/1040) participants did not respond to any postenrollment surveys or provide passive data and were therefore considered to be study dropouts (Figure 3). Income, education, and race were significantly different between those who dropped and those who did not (*P*<.005; Multimedia Appendix 1). A large proportion of individuals who reported that they “can’t make ends meet” with regard to their income dropped out of the study (238/692, 34.4%) this effect was more pronounced for Hispanic/Latino individuals (135/283, 47.7%). Over half (171/283, 60.4%) of the Hispanic/Latino participants who dropped out of the study reported making US $20,000 or less annually compared to with 28.10% (112/398) of non-Hispanic/Latinos who dropped out. Of the 348 active individuals, 74 did not complete the treatment randomization survey and thus were not assigned an intervention. However, they continued to complete self-report surveys during the study period. For this reason, we categorized these participants as enrolled but not randomized (EnR) category. All further analyses were restricted to active individuals consisting of those in treatment (n=274) or EnR (n=74; total N=348) arms. See Figure 3 for the Consolidated Standards of Reporting Trials diagram illustrating participant flow through the study.

Of those who were randomized, 31.8% (87/274) attempted to change their assigned intervention by hitting the “back” button to return to the randomization page, while an additional 10.2% (28/274) participants returned to the survey a second time to change their preferences (9/274, 3.2%) of these individuals used both methods). Note that these attempts were unsuccessful because participant randomization was determined by the first answer given by a participant, and not any of the subsequent attempts made.

### Sample Demographics

See Table 1 for participant characteristics, including comparisons across those identifying as Hispanic/Latino and not. The participants were predominantly young, with 69.81% (238/345) aged less than 40 years (mean 34.90, SD 10.92); female (205/266, 77.19%); and non-Hispanic white (98/184, 53.3%), with 30.7% (33/106) of our sample reporting Hispanic/Latino identity. The majority (168/241, 69.9%) reported some form of employment, and 87.8% (266/303) of all participants were iPhone users. There were significant differences between Hispanic/Latino and non-Hispanic/Latino participants; notably, a greater proportion (43/106, 40.6%) of Hispanic/Latino participants reported annual incomes of less than US $20,000, compared with only 24.7% (59/239) non-Hispanic/Latinos. Likewise, non-Hispanic/Latino participants were significantly more likely to be employed and more likely to have obtained a university education relative to Hispanic/Latino participants. Finally, Hispanic/Latino participants were slightly younger than their counterparts, although both groups were on average in their early-to-mid 30s.

### Clinical Characteristics

**Figure 4:**
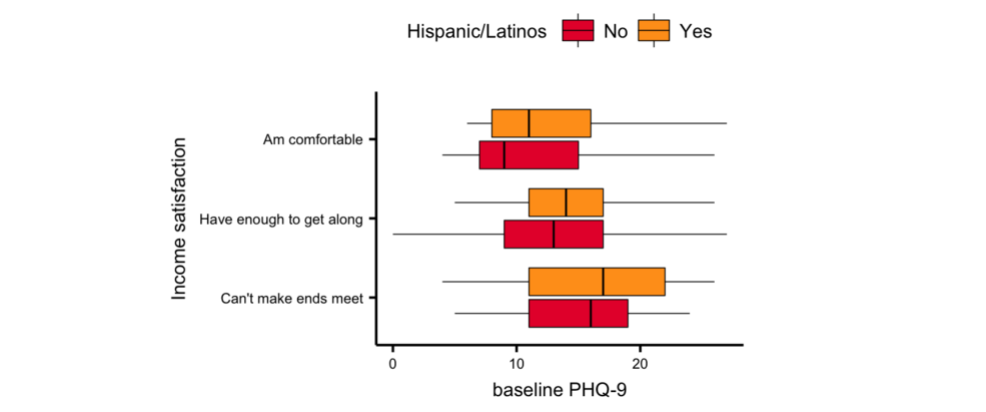
Comparison of self-reported income satisfaction and baseline Patient Health Questionnaire-9 (PHQ-9) score between Hispanic/Latino and non-Hispanic/Latino participants.

Overall, the cohort reported moderate depressive symptomatology with a mean baseline PHQ-9 of 13.61 (SD 5.46). There was no difference in baseline depression between Hispanic/Latino and non-Hispanic/Latino participants (*P*=.07), and neither age nor gender showed a significant association with baseline PHQ-9 scores (age: ⍴=−0.09, *P*=.06; gender: *F*_1336_=3.16, *P*=.07). Income satisfaction showed a moderate effect on baseline PHQ-9 scores (*f*^2^=0.265, *P*<.001). Table 2 summarizes the associations and effect sizes of all baseline variables with baseline PHQ-9 scores. Participants who reported income satisfaction as “can’t make ends meet” showed significantly higher depression symptomatology (delta PHQ-9=+3.9, *P*<.001) than those who reported income level as “comfortable” (Figure 4). However, this discrepancy in depressive symptoms between income levels was not significantly different between Hispanic/Latinos and non-Hispanic/Latinos across other categories of income satisfaction.

**Table 1:** BRIGHTEN V2 participant characteristics.

Characteristics		Overall^a^ (N=345)	Hispanic/Latino (n=106)	Non-Hispanic/Latino (n=239)		*P* value	
Baseline Patient Health Questionnaire-9, mean (SD)		13.61 (5.46)	14.41 (5.69)	13.26 (5.34)			.08
Gender (female), n (%)		266 (77.1)	82 (77.4)	184 (77.0)			>.99
Age (years), mean (SD)		34.90 (10.92)	32.71 (10.10)		35.88 (11.15)		.02
**Age (years), n (%)**							.22
	18-30	137 (40.2)	51 (48.6)		86 (36.4)		
	31-40	101 (29.6)	27 (25.7)		74 (31.4)		
	41-50	74 (21.7)	22 (21.0)		52 (22.0)		
	51-60	23 (6.7)	5 (4.8)		18 (7.6)		
	61-70	5 (1.5)	0 (0.0)		5 (2.1)		
	>70	1 (0.3)	0 (0.0)		1 (0.4)		
**Income last year (US $), n (%)**							.005
	20,000 or less	102 (29.6)	43 (40.6)		59 (24.7)		
	20,000-40,000	90 (26.1)	31 (29.2)		59 (24.7)		
	40,000-60,000	76 (22.0)	20 (18.9)		56 (23.4)		
	60,000-80,000	32 (9.3)	5 (4.7)		27 (11.3)		
	80,000-100,000	22 (6.4)	2 (1.9)		20 (8.4)		
	100,000	23 (6.7)	5 (4.7)		18 (7.5)		
**Education, n (%)**							<.001
	Community college	72 (20.9)	25 (23.6)		47 (19.7)		
	Graduate degree	58 (16.8)	11 (10.4)		47 (19.7)		
	High school	56 (16.2)	29 (27.4)		27 (11.3)		
	University	159 (46.1)	41 (38.7)		118 (49.4)		
Device (iPhone), n (%)		303 (87.8)	89 (84.0)		214 (89.5)		.20
Working (Yes), n (%)		241 (69.9)	65 (61.3)		176 (73.6)		.03
**Race, n (%)**							<.001
	Hispanic/Latinos	106 (30.7)	106 (100.0)		0 (0.0)		
	Non-Hispanic white	184 (53.3)	0 (0.0)		184 (77.0)		
	African-American/black	25 (7.2)	0 (0.0)		25 (10.5)		
	American Indian/Alaskan Native	3 (0.9)	0 (0.0)		3 (1.3)		
	Asian	24 (7.0)	0 (0.0)		24 (10.0)		
	Other	3 (0.9)	0 (0.0)		3 (1.3)		
Speak Spanish (yes), n (%)		113 (32.8)	96 (90.6)		17 (7.1)		<.001
**Income satisfaction, n (%)**							.09
	Comfortable	71 (20.6)	17 (16.0)		54 (22.6)		
	Can't make ends meet	80 (23.2)	32 (30.2)		48 (20.1)		
	Have enough to get along	194 (56.2)	57 (53.8)		137 (57.3)		
**Marital status, n (%)**							.28
	Married/Partnered	135 (39.1)	35 (33.0)		100 (41.8)		
	Separated/Widowed/Divorced	33 (9.6)	12 (11.3)		21 (8.8)		
	Single	177 (51.3)	59 (55.7)		118 (49.4)		

^a^Participants who did not self-report Hispanic/Latinos status (n=3) have not been compared.

**Table 2:** Association between baseline demographic variables and Patient Health Questionnaire-9 scores.

Baseline variables	Cohen *f*^2^	False Discovery Rate
Income satisfaction	0.264	<0.001
Income	0.226	0.02
Spanish speaker	0.139	0.029
Education	0.160	0.076
Working	0.103	0.096
Hispanic/Latinos	0.098	0.101
Marital status	0.107	0.15
Race	0.161	0.15

### Cost

Study costs beyond the initial infrastructure developed for BRIGHTEN V1 included participant payments (US $7540), website/enrollment portal/database development (US $4601), and total recruitment efforts (US $14,471; see Table 3). A bulk of recruitment spending was for 217 Spanish language ads placed on Craigslist throughout the country (US $5725), while only US $946 was spent on 33 English ads to obtain the reported enrollment. Furthermore, US $7800 was spent on targeted social media recruitment specifically for Spanish speakers via Trialspark.com; however, only 86 unique registrants came through this portal. Thus, participant acquisition costs differed dramatically between Spanish (US $31 per enrolled participant) and English speakers (US $1.49 per enrolled participant).

### Engagement

**Figure 5:**
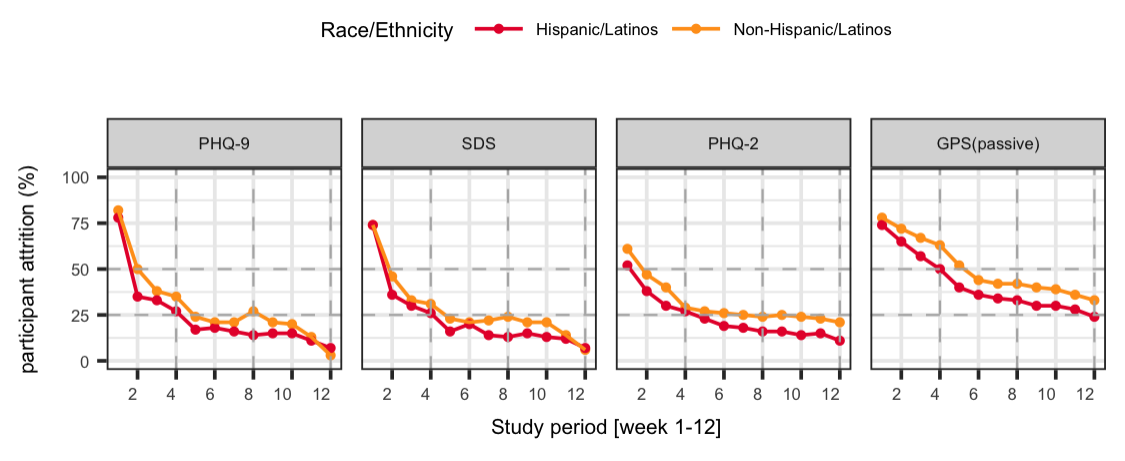
Comparison of participant attrition in the study across survey types and passive data stratified by Hispanic/Latinos and Non-Hispanic/Latinos. GPS: Global Positioning System; PHQ-2: 2-item Patient Health Questionnaire; PHQ-9: 9-item Patient Health Questionnaire; SDS: Sheehan Disability Scale.

**Figure 6:**
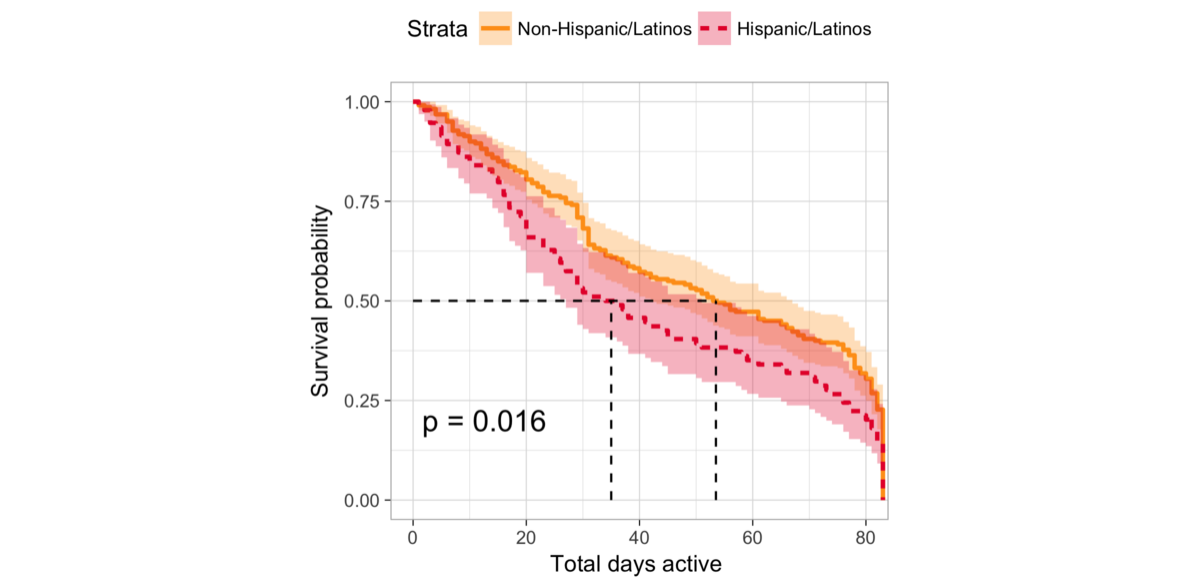
Comparison of Kaplan-Meier survival estimates for Hispanic/Latino and non-Hispanic/Latino participants during the course of the study (1-84) days.

**Figure 7:**
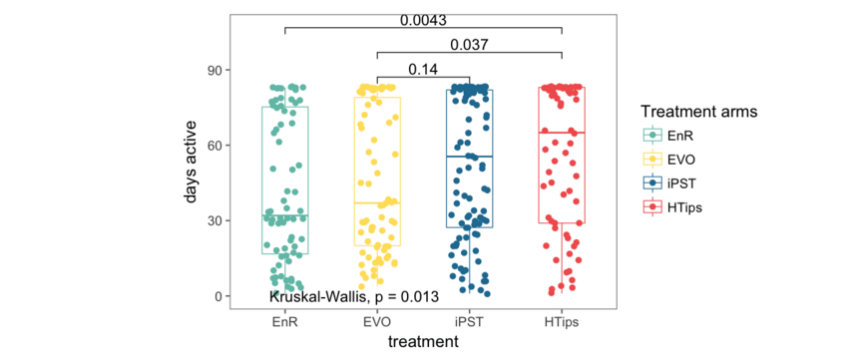
Comparison of number of days participants were active across different treatment arms in the study. EnR: enrolled but not randomized; EVO: Project Evolution; HTips: health tips; iPST: internet-based problem-solving therapy.

Overall participation in the study (as measured by assessment completion, as opposed to intervention app use) decreased by approximately 50% from week 1 to week 4, with more than 4 out of 5 participants dropping (14%) out by the end of 12 weeks. At week 4, participants contributed twice as much passive data (ie, momentary Global Positioning System data) compared with that provided in survey assessments requiring active participation (Figure 5). Significant differences in participant engagement were observed between Hispanic/Latino and non-Hispanic/Latino participants (*P*=.02). Non-Hispanic/Latino individuals tended to participate in the study for 18.5 days longer than their Hispanic/Latino counterparts (median 53.5 days until dropout for non-Hispanic/Latinos and median 37 for Hispanic/Latino participants; see Figure 6)*.* Finally, participants in the iPST and HTips arms were significantly more engaged than those in the EVO and EnR arms (*P*<.01), regardless of the race/ethnicity (Figure 7).

### Depression Outcomes

**Figure 8:**
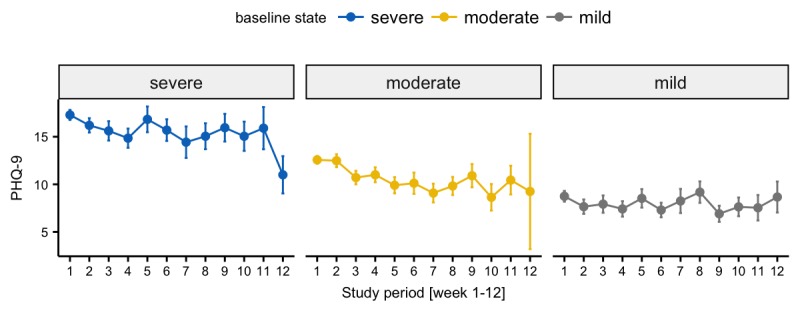
Comparison of weekly mean Patient Health Questionnaire-9 (PHQ-9) scores with mean SEs stratified by baseline depression state.

Changes in weekly PHQ-9 scores were significantly associated with baseline severity of depressive symptoms (ie, mild, moderate, and severe; *P*<.001). Participants who reported severe depressive symptoms upon study entry evidenced the greatest decline in PHQ-9 scores during weeks 1-4 (beta=−4.19, *P*<.001) but no significant changes during weeks 5-12. Participants with moderate symptoms also showed an initial decline in PHQ-9 (beta=−1.96, *P*=.004) and a further decline of 0.70 points (beta=−2.66, *P*=.006) in weeks 5-12 (Table 4,Figure 8). With regard to treatment remission at the end of week 4, 34.42% participants responded to the interventions (a decrease in PHQ-9 score of ≥5 from baseline), 51.63% were nonresponders (change in PHQ-9 of <5 points), and a small proportion (11.48%) deteriorated (PHQ-9 worsened ≥5 points) during the course of the study. However, there was no difference in depression outcomes among the 3 intervention arms. No differences in treatment remission were observed between Hispanic/Latino participants and non-Hispanic/Latinos.

### Disability Outcomes

At the cohort level, disability based on SDS ratings decreased by an average 0.74 points (*P*=.03) in weeks 2-4 and further by 0.39 points (beta=−1.09, *P*=.02) in weeks 5-12. As with depression outcomes, there was no difference in disability outcomes across treatment arms. Hispanic/Latino and non-Hispanic/Latino participants did not differ in their disability outcomes (Table 5).

**Table 3:** Participant acquisition costs.

Recruitment approach	Amount spent (US $)	Participants reached, n	Cost per participant (US $)
Targeted Social Media (trialspark.com for Spanish Speakers)	7800	86	90.70
Craigslist.com (Spanish advertisements)	5275	303	17.41
Craigslist.com (English advertisements)	946	637	1.49

**Table 4:** Summary of estimates comparing weekly change in Patient Health Questionnaire-9 scores using a generalized estimating equations model.

Fixed effects	Effect size, beta (SE)	*P* value
Intercept	8.28 (0.77)	<.001
Gender (male)	.09 (0.50)	.85
Age	−.02 (0.02)	.23
Weeks 1-4	1.33 (0.55)	.02
Weeks 5-12	1.33 (0.72)	.06
Treatment (EVO^a^)	.03 (0.57)	.96
Treatment (HTips^b^)	−.93 (0.56)	.09
Treatment (iPST^c^)	−.39 (0.53)	.45
Hispanic/Latinos (yes)	−0.15 (0.43)	.73
Baseline state (moderate)	5.35 (0.39)	<.001
Baseline state (severe)	12.26 (0.46)	<.001
Weeks 1-4: baseline state (moderate)	−1.96 (0.67)	.004
Weeks 5-12: baseline state (moderate)	−2.66 (0.96)	.006
Weeks 1-4: baseline state (severe)	−4.19 (0.77)	<.001
Weeks 5-12: baseline state (severe)	−4.31 (1.04)	<.001

^a^EVO: Project Evolution.

^b^HTips: health tips.

^c^iPST: internet-based problem-solving therapy.

**Table 5:** Summary of estimates comparing weekly change in Sheehan Disability Scale score using a generalized estimating equations model.

Fixed effects	Effect size, beta (SE)	*P* value
Intercept	10.91 (1.61)	<.001
Gender (male)	.64 (0.85)	.46
Age	.00 (0.04)	.89
Treatment (EVO^a^)	.32 (1.14)	.78
Treatment (HTips^b^)	−.74 (1.07)	.49
Treatment (iPST^c^)	−.12 (1.04)	.91
Weeks 2-4	−.70 (0.33)	.03
Weeks 5-12	−1.09 (0.47)	.02
Hispanic/Latinos (yes)	.12 (0.82)	.88

^a^EVO: Project Evolution.

^b^HTips: health tips.

^c^iPST: internet-based problem-solving therapy.

## Discussion

### Principal Findings

To our knowledge, BRIGHTEN V2 is the first large-scale effort to target the remote recruitment of Hispanic/Latino individuals with depression in the United States using digital health assessments and interventions that were translated into Spanish and administered solely on smartphones. We screened and enrolled one of the largest cohorts of Hispanic/Latino individuals with depression to date. Previous work has suggested that the lack of utilization of mental health care could be attributed to (1) cultural beliefs about mental health problems, (2) ineffective and inappropriate therapies, or (3) access problems or other barriers [24]. We attempted to address each of these issues by selectively targeting an underrepresented Hispanic/Latino population and using accessible, Spanish translated versions of the evidence-based intervention apps used in the initial study [8]. As has been found in other mobile-based mental health clinical trials [25,26], long-term engagement continues to be a significant challenge to these studies and is more pronounced among Hispanic/Latino participants. Although mobile devices are increasingly available in Hispanic/Latino communities [10], the availability of these devices as a means for conducting research and delivering care are not yet solutions that offset the widespread disparities seen in this population.

### Feasibility and Acceptability

Similar to our previous work [7,27], this study has shown the feasibility of recruiting and enrolling a large and diverse sample of Hispanics/Latino adults. Previous research and observations from clinical practice suggest that Hispanics/Latino populations in the United States face barriers to research and treatment, including stigma and time constraints. This study was intended to overcome those very barriers by leveraging mobile apps that could be used at each participant’s convenience. However, the engagement data showed that the Hispanics/Latino participants dropped out close to 2 weeks earlier than their non-Hispanics/Latinos counterparts, highlighting significant challenges in not only recruiting but also in keeping this population engaged. It was much more expensive and labor intensive to recruit Hispanics/Latino participants relative to the rest of the cohort. Attrition was particularly striking among the Hispanic/Latino subset, with only 18.7% (73/389) downloading the treatment app. Highest dropout among the Hispanic/Latino sample were from participants reporting an annual income level of less than US $20,000.

Potential issues recruiting US Hispanic/Latino individuals for mental health research may hinge on (1) reluctance to be randomized, given the high number of the enrolled participants who tried to switch the initial randomly assigned intervention app and (2) privacy concerns such as the possibility that some of our lower income participants could be sharing the smartphones with other family members, potentially reducing the willingness to participate and causing high initial dropout [28]. Furthermore, the majority of participants were iPhone users, which may not be representative of the underlying population. While the ownership of an Android smartphone plus an iPad combination is relatively common as indicated by a 2014 survey [9], the ease of being able to participate in this study by only having to have a single device (iOS phone) likely spurred the bias toward iOS users in the sample.

Another potential issue in the study was the possible delay in receiving the intervention. The stratified equipoise randomization occurred after eligible participants attempted the assigned assessments (or after 72 hours, whichever came first); given that participants may have been waiting for their assigned intervention following their initial exposure to the assessment app, they may have lost interest in participating. Another consideration involves the appropriate incentive structure (eg, timing and amount of compensation) to maximize retention and engagement, as this factor is not well understood among such underrepresented samples such as ours. It is an empirical question to understand how the amount of payment affects one’s participation in a given trial. Indeed, in the first version of this study (BRIGHTEN V1), we found that participants who received bonus payments remained in the study longer than those who did not receive bonuses [8]. In that study, the experimentation with two distinct incentive models to encourage retention revealed that participant payment was not enough to keep engagement from waning. Other work has shown that externalized benefits (eg, compensation) can dull motivation, whereas the creation of an internalized reward structure can enhance motivation and improve the aspects of adherence (eg, individualized presentation of study progress, personalized encouragements) [29,30]. This is a considerable hurdle to overcome for mental health researchers who are dependent upon trying to identify features that would align with greater engagement of a culturally unique population. Thus, these issues of acceptability and engagement must be dealt with not only for research but also for any scalable intervention to take hold in routine clinical practice.

Despite the poor engagement of the active components in this study, it is clear from the findings (and those from other mobile-based studies) that there is still a tremendous potential to capture passive data from smartphone use. This form of data capture is much less burdensome as it does not require the user to actively engage with an app. If one only considers the passive data compliance versus that of the active surveys in our study, passive data offers a viable opportunity to develop an individualized digital baseline (digital fingerprint) and investigate deviations from baseline phone usage to behavioral fluctuations. However, using cohort-level signals in passive data to predict depression states remains modest at best [31-33], suggesting that this approach will likely require larger studies and pairing with an active task-based component for the most effective solution.

### Difference in Clinical Features and Outcomes

Similar to our earlier findings in the original study [7], participants on average reported improvement in both depression and disability measures over time, regardless of treatment arm. However, more than half of the participants, regardless of their race/ethnicity, did not evidence any clinically meaningful change (PHQ-9 change of less than 5 points from baseline) or actually deteriorated according to their PHQ-9 scores (worsening of more than 5 points from baseline on PHQ-9) during the course of the study. It is important to note that the participants in our trial did not have a clinical diagnosis of depression, rather they endorsed at least a mild level of depressive symptomatology at baseline screening on PHQ-9. Moreover, treatment outcomes were based on self-report using this screening measure. Perhaps unsurprisingly, treatment response was strongest in those with greater depressive symptomatology at baseline. Thus, we interpret our clinical findings with caution, as this is not a clinical sample or an effectiveness trial, but rather a feasibility trial in a sample of potential interest for future remote interventions. We also noted overall poor engagement in this sample with significant demographic differences between our Hispanic/Latino and non-Hispanic/Latino participants. Hispanic/Latinos reported lower income, less income satisfaction, and lower education; such factors have been previously reported to be associated with an increased incidence of depression [34].

### Conclusions and Future Directions

mHealth platforms have the potential to deliver on-demand and as needed assessment and intervention alternatives despite known barriers of time constraints, cost, stigma, and cultural and language differences. Although mHealth holds great promise for closing the treatment gap for underserved communities, recruitment and retention remain problematic in such populations, and more research is needed to figure out better engagement strategies to best leverage mobile apps (eg, appropriate incentive levels, culturally responsive content and notifications along with user-centered design approaches [35]). Like other contactless programs (eg, self-help interventions), it is difficult to keep users engaged in active components without therapists or other in-person supports [36]. However, the ubiquity and relative unobtrusive nature of smartphones lend itself to acquiring passive sensing data, even in the absence of engagement with active components of the research or intervention protocol.

Our study offers preliminary lessons learned from doing such work in an understudied sample of Hispanic/Latino smartphone users. Scaling these types of remote assessments and interventions will hinge on the acceptance of such technology by both care teams and patients. This will be a problem for future research using remote technologies at scale to recruit and engage targeted communities (eg, Hispanic/Latino adults with depression) and will depend on understanding the population’s needs and addressing barriers to using mental health interventions via mobile apps.

## Appendix

Multimedia Appendix 1 Comparison of demographic variables.

Multimedia Appendix 2 CONSORT‐EHEALTH checklist (V 1.6.1).

## Abbreviations

GEE: generalized estimating equations
EnR: enrolled but not randomized
EVO: Project Evolution
HTips: health tips
iPST: internet-based problem-solving therapy
PHQ-9: Patient Health Questionnaire-9
SDS: Sheehan Disability Scale

## References

[1] (2018). Pew Research Center.

[2] Olfson M, Blanco C, Marcus SC (2016). Treatment of Adult Depression in the United States. JAMA Intern Med.

[3] Arevalo M, Heredia N, Krasny S, Rangel M, Gatus L, McNeill L, Fernandez Maria E (2016). Mexican-American perspectives on participation in clinical trials: A qualitative study. Contemp Clin Trials Commun.

[4] Miranda J, Nakamura R, Bernal G (2003). Including Ethnic Minorities in Mental Health Intervention Research: A Practical Approach to a Long-Standing Problem. Cult Med Psychiatry.

[5] Fairburn C, Patel V (2017). The impact of digital technology on psychological treatments and their dissemination. Behav Res Ther.

[6] Carlbring P, Andersson G, Cuijpers P, Riper H, Hedman-Lagerlöf Erik (2018). Internet-based vs. face-to-face cognitive behavior therapy for psychiatric and somatic disorders: an updated systematic review and meta-analysis. Cogn Behav Ther.

[7] Arean PA, Hallgren KA, Jordan JT, Gazzaley A, Atkins DC, Heagerty PJ, Anguera JA (2016). The Use and Effectiveness of Mobile Apps for Depression: Results From a Fully Remote Clinical Trial. J Med Internet Res.

[8] Anguera J, Jordan J, Castaneda D, Gazzaley A, Areán Patricia A (2016). Conducting a fully mobile and randomised clinical trial for depression: access, engagement and expense. BMJ Innov.

[9] Dorsey E, Yvonne CY, McConnell M, Shaw S, Trister A, Friend S (2017). The Use of Smartphones for Health Research. Acad Med.

[10] Lavori P, Rush A, Wisniewski S, Alpert J, Fava M, Kupfer D, Nierenberg A, Quitkin F M, Sackeim H A, Thase M E, Trivedi M (2001). Strengthening clinical effectiveness trials: equipoise-stratified randomization. Biol Psychiatry.

[11] Löwe Bernd, Unützer Jürgen, Callahan C, Perkins A, Kroenke K (2004). Monitoring depression treatment outcomes with the patient health questionnaire-9. Med Care.

[12] Leon A, Olfson M, Portera L, Farber L, Sheehan D (1997). Assessing psychiatric impairment in primary care with the Sheehan Disability Scale. Int J Psychiatry Med.

[13] Kroenke K, Spitzer R, Williams J (2001). The PHQ-9: validity of a brief depression severity measure. J Gen Intern Med.

[14] Final Recommendation Statement: Depression in Adults: Screening - US Preventive Services Task Force Internet.

[15] Wulsin L, Somoza E, Heck J (2002). The Feasibility of Using the Spanish PHQ-9 to Screen for Depression in Primary Care in Honduras. Prim Care Companion J Clin Psychiatry.

[16] Sheehan K, Sheehan D (2008). Assessing treatment effects in clinical trials with the discan metric of the Sheehan Disability Scale. Int Clin Psychopharmacol.

[17] Bobes J, Badía X, Luque A, García M, González M P, Dal-Ré R (1999). [Validation of the Spanish version of the Liebowitz social anxiety scale, social anxiety and distress scale and Sheehan disability inventory for the evaluation of social phobia]. Med Clin (Barc).

[18] Anguera J, Gunning F, Areán Patricia A (2017). Improving late life depression and cognitive control through the use of therapeutic video game technology: A proof-of-concept randomized trial. Depress Anxiety.

[19] Liang K, Zeger S (1986). Longitudinal Data Analysis Using Generalized Linear Models. Biometrika.

[20] Ballinger G (2004). Using Generalized Estimating Equations for Longitudinal Data Analysis. Organizational Research Methods.

[21] Rich J, Neely J, Paniello R, Voelker C, Nussenbaum B, Wang E (2010). A practical guide to understanding Kaplan-Meier curves. Otolaryngol Head Neck Surg.

[22] Bland J (2004). The logrank test. BMJ.

[23] The R Project for Statistical Computing Internet.

[24] Vega W, Kolody B, Aguilar-Gaxiola S, Catalano R (1999). Gaps in service utilization by Mexican Americans with mental health problems. Am J Psychiatry.

[25] Miranda J, Azocar F, Organista K, Muñoz R F, Lieberman A (1996). Recruiting and retaining low-income Latinos in psychotherapy research. J Consult Clin Psychol.

[26] Brown G, Marshall M, Bower P, Woodham A, Waheed W (2014). Barriers to recruiting ethnic minorities to mental health research: a systematic review. Int J Methods Psychiatr Res.

[27] Arean PA, Hallgren KA, Jordan JT, Gazzaley A, Atkins DC, Heagerty PJ, Anguera JA (2016). The Use and Effectiveness of Mobile Apps for Depression: Results From a Fully Remote Clinical Trial. J Med Internet Res.

[28] Karlson A, Brush A, Schechter S (2009). Can i borrow your phone?.

[29] Cruz M, Pincus H, Harman J, Reynolds C3, Post E (2008). Barriers to care-seeking for depressed African Americans. Int J Psychiatry Med.

[30] Van ED (2006). Psychotherapy with older adults: benefits and barriers. J Psychosoc Nurs Ment Health Serv.

[31] Saeb S, Zhang M, Karr C, Schueller S, Corden M, Kording K, Mohr David C (2015). Mobile Phone Sensor Correlates of Depressive Symptom Severity in Daily-Life Behavior: An Exploratory Study. J Med Internet Res.

[32] Saeb S, Lattie EG, Schueller SM, Kording KP, Mohr DC (2016). The relationship between mobile phone location sensor data and depressive symptom severity. PeerJ.

[33] Pratap A, Anguera J, Renn B, Neto E, Volponi J, Mooney S (2017). The feasibility of using smartphones to assess and remediate depression in Hispanic/Latino individuals nationally.

[34] Lorant V, Deliège D, Eaton W, Robert A, Philippot P, Ansseau M (2003). Socioeconomic inequalities in depression: a meta-analysis. Am J Epidemiol.

[35] Vredenburg K, Mao J, Smith P, Carey T (2002). A survey of user-centered design practice.

[36] Aguilera A (2015). Digital Technology and Mental Health Interventions: Opportunities and Challenges. Arbor.

