# Supplementary material for: Using Mobile Apps to Assess and Treat Depression in Hispanic and Latino Populations: Fully Remote Randomized Clinical Trial
Source: J Med Internet Res. 2018 Aug 9;20(8):e10130. doi: 10.2196/10130 (PMC6107735; doi:10.2196/10130)
Supplement: Multimedia Appendix 1 [file jmir_v20i8e10130_app1.pdf]

Multimedia Appendix 1

Using Mobile Apps to Assess and Treat Depression in Hispanics and Latinos: Fully Remote Randomized Clinical Trial

Table 1 - Comparison of demographic variables between active and dropped-out participants...............2
Table 2 - Comparison of demographic variables between Hispanic/Latinos and Non-Hispanic/Latinos further stratified by Active and Dropped-out participants...4
Recruiting Approaches...5
Spanish Craigslist Ad..5
Approaches taken to ensure quality of data..7
Data Acquisition Parameters..8
Adaptive Cognitive Evaluation for Mental Health (ACEmh)...8

Table 1 - Comparison of demographic variables between active and dropped-out participants

	**Active participants***	**Dropped-out***	***p-value***
**N(%)**	345 (33.62)	681 (66.37)	
**Baseline PHQ-9 score (mean (sd))**	13.61 (5.46)	14.80 (5.51)	**0.001**
**Suicidal Ideation**	85 (24.63)	245 (35.97)	**< .001**
**Gender = Female (%)**	266 (77.1)	472 (69.3)	**0.011**
**Age(mean (sd))**	34.90 (10.92)	36.75 (75.75)	0.652
**Age group(%)**			0.685
18-30	137 (40.2)	298 (44.2)	
31-40	101 (29.6)	202 (30.0)	
41-50	74 (21.7)	123 (18.2)	
51-60	23 (6.7)	38 (5.6)	
61-70	5 (1.5)	12 (1.8)	
70+	1 (0.3)	1 (0.1)	
**Device= iPhone (%)**	303 (87.8)	568 (83.4)	0.076
**Working= Yes (%)**	241 (69.9)	446 (65.5)	0.182
**Race (%)**			**0.004**
Hispanic/Latino	106 (30.7)	284 (41.7)	
Non-hispanic White	184 (53.3)	301 (44.2)	
African-American/Black	25 (7.2)	56 (8.2)	
American Indian/Alaskan Native	3 (0.9)	9 (1.3)	
Asian	24 (7.0)	25 (3.7)	
Other	3 (0.9)	6 (0.9)	
**Speak Spanish = Yes (%)**	113 (32.8)	289 (42.4)	**0.003**
**Income last year (%)**			**<0.001**
$20,000 or less	102 (29.6)	283 (41.6)	
20,000-40,000	90 (26.1)	165 (24.2)	
40,000-60,000	76 (22.0)	96 (14.1)	
60,000-80,000	32 (9.3)	40 (5.9)	
80,000-100,000	22 (6.4)	24 (3.5)	
100,000+	23 (6.7)	73 (10.7)	
**Income satisfaction (%)**			**0.001**
Am comfortable	71 (20.6)	109 (16.0)	
Can't make ends meet	80 (23.2)	234 (34.4)	
Have enough to get along	194 (56.2)	338 (49.6)	
**Marital status (%)**			0.279
Married/Partner	135 (39.1)	262 (38.5)	
Separated/Widowed/Divorced	33 (9.6)	88 (12.9)	
Single	177 (51.3)	331 (48.6)	
**Education (%)**			**<0.001**

University	159 (46.1)	261 (38.3)	
High School	56 (16.2)	195 (28.6)	
Community College	72 (20.9)	144 (21.1)	
Elementary School	0 (0.0)	6 (0.9)	
Graduate Degree	58 (16.8)	75 (11.0)	

Table 2 - Comparison of demographic variables between Hispanic/Latinos and Non-Hispanic/Latinos further stratified by Active and Dropped-out participants

	**Hispanic/Latinos**		**Non-Hispanic/Latinos**		
	**Active**	**Dropped-out**	**Active**	**Dropped-out**	***p-value***
**N (%)**	106	283	239	398	
**Baseline PHQ-9 (mean (sd))**	14.41 (5.69)	14.82 (5.62)	13.26 (5.34)	14.78 (5.43)	0.004
**Suicidal Ideation**	28 (32.6)	93(43.3)	57(24.5)	152(38.9)	**<0.001**
**Gender = Female (%)**	82 (77.4)	216 (76.3)	184 (77.0)	256 (64.3)	**<0.001**
**Age(mean (sd))**	32.71 (10.10)	40.05 (116.82)	35.88 (11.15)	34.41 (10.97)	0.624
**Age group(%)**					0.395
18-30	51 (48.6)	123 (44.2)	86 (36.4)	175 (44.2)	
31-40	27 (25.7)	86 (30.9)	74 (31.4)	116 (29.3)	
41-50	22 (21.0)	55 (19.8)	52 (22.0)	68 (17.2)	
51-60	5 (4.8)	9 (3.2)	18 (7.6)	29 (7.3)	
61-70	0 (0.0)	5 (1.8)	5 (2.1)	7 (1.8)	
70+	0 (0.0)	0 (0.0)	1 (0.4)	1 (0.3)	
**device = iPhone (%)**	89 (84.0)	235 (83.0)	214 (89.5)	333 (83.7)	0.15
**Working = Yes (%)**	65 (61.3)	149 (52.7)	176 (73.6)	297 (74.6)	**<0.001**
**Race (%)**					**<0.001**
Hispanic_Latino	106 (100.0)	283 (100.0)	0 (0.0)	1 (0.3)	
Non-hispanic White	0 (0.0)	0 (0.0)	184 (77.0)	301 (75.6)	
African-American/Black	0 (0.0)	0 (0.0)	25 (10.5)	56 (14.1)	
American Indian/Alaskan Native	0 (0.0)	0 (0.0)	3 (1.3)	9 (2.3)	
Asian	0 (0.0)	0 (0.0)	24 (10.0)	25 (6.3)	
Other	0 (0.0)	0 (0.0)	3 (1.3)	6 (1.5)	
**Speak Spanish = Yes (%)**	96 (90.6)	257 (90.8)	17 (7.1)	32 (8.0)	**<0.001**
**Income lastyear (%)**					**<0.001**
$20,000 or less	43 (40.6)	171 (60.4)	59 (24.7)	112 (28.1)	
20,000-40,000	31 (29.2)	63 (22.3)	59 (24.7)	102 (25.6)	
40,000-60,000	20 (18.9)	30 (10.6)	56 (23.4)	66 (16.6)	
60,000-80,000	5 (4.7)	11 (3.9)	27 (11.3)	29 (7.3)	
80,000-100,000	2 (1.9)	3 (1.1)	20 (8.4)	21 (5.3)	
100,000+	5 (4.7)	5 (1.8)	18 (7.5)	68 (17.1)	
**Income satisfaction (%)**					**<0.001**
Am comfortable	17 (16.0)	47 (16.6)	54 (22.6)	62 (15.6)	
Can't make ends meet	32 (30.2)	135 (47.7)	48 (20.1)	99 (24.9)	
Have enough to get along	57 (53.8)	101 (35.7)	137 (57.3)	237 (59.5)	
**Marital status (%)**					**0.016**
Married/Partner	35 (33.0)	101 (35.7)	100 (41.8)	161 (40.5)	
Separated/Widowed/Divorced	12 (11.3)	50 (17.7)	21 (8.8)	38 (9.5)	
Single	59 (55.7)	132 (46.6)	118 (49.4)	199 (50.0)	
**Education (%)**					**<0.001**

Community College	25 (23.6)	69 (24.4)	47 (19.7)	75 (18.8)	
Elementary School	0 (0.0)	5 (1.8)	0 (0.0)	1 (0.3)	
Graduate Degree	11 (10.4)	21 (7.4)	47 (19.7)	54 (13.6)	
High School	29 (27.4)	100 (35.3)	27 (11.3)	95 (23.9)	
University	41 (38.7)	88 (31.1)	118 (49.4)	173 (43.5)	

Recruiting Approaches

Of the different recruitment strategies used, Craigslist provided the greatest response rate.

1) **Traditional recruitment:** consisted of outreach to provider networks, print advertisements and flyers placed in strategic locations, such as coffee shops, libraries and bus stops across the city of San Francisco, as especially online classified pages like craigslist (see eFigure 1). Ads specified that UCSF was conducting a study to better understand the effects of mobile apps for one's well being, and was seeking volunteers. A web-link to the study portal was provided in the ads. Craigslists ads were placed in the largest cities in each state under volunteer opportunity as well as under 'Jobs etc./part-time jobs'.

Spanish Craigslist Ad

¿Se siente triste o deprimido? Un investigación usando su teléfono

¿Tiene más de 18 años? ¿A veces se siente deprimido? ¿Tiene problemas para concentrarse? ¿Tienes un iPhone o ANDROID?

El objetivó de este investigación: Científicos del Departamento de Psiquiatría y Neurología de la Universidad de California, San Francisco (UCSF) quieren evaluar la utilidad de las aplicaciones móviles para mejorar el estado de ánimo y la concentración.

Si usted es elegible:

-Si calificas, vas a utilizar un aplicación móvil por un período de cuatro semanas, y completando algunas preguntas en su teléfono por 12 semanas en total.
- Sobre el curso del estudio, se le pedirá que complete las encuestas de evaluación sobre su estado de ánimo
- Vas ha hacer todo para este estudio (evaluación y tratamiento) en su iPhone o ANDROID.
- Si eres elegible, pueden recibir hasta $ 75.
- Las aplicaciones utilizadas en este estudio se consideran un programa de "auto-ayuda " , y no se consideran un sustituto del tratamiento activo.

Por favor vaya a https://trialspark.com/trials/brightenstudy para registrar

La compensación es en tarjetas/códigos electrónicas de regalo de Amazon. Dependiendo en la cantidad del estudio que se completa, puedes ganar hasta $ 75 en total !

Tarjetas de regalo de Amazon. Dependiendo en la cantidad del estudio que se completa, puedes ganar hasta $ 75 en total !

2) **Social Networking:** Social network-based recruitment consisted of social media lead advertising. Through our partner TrialSpark.com, machine learning-based approaches were used to create "Intelligent lead generation" to promote our study to users on Twitter. This involved the creation of

custom messaging and triaging of key words that indicated they may be suffering from symptoms of depression. The potential participants were forwarded one of several similar brief advertisements making them aware of the BRIGHTEN study, with a link to the study website. eFigure 2 shows an example custom message used by TrialSpark.com recruitment

¡Hola! Muchas Gracias por su interés en nuestra investigación.
Estamos reclutando participantes interesados ahora para el lanzamiento de nuestro estudio de este año.
Anticipamos que eso será en Mayo del 2016…tan pronto!
Usted será contactado por correo electrónico el día que el estudio comienza oficialmente.
Gracias de nuevo por su paciencia, estamos muy contentos de trabajar con usted en este proyecto!

3) **Search engine-based recruitment** consisted of strategically placed ads, similar to ads that appear in usual media recruitment, on search engines and social networking communities (e.g., Google Adwords). The ads that will appear via this method are similar to the traditional ads, with a direct link to the study portal provided on the ads

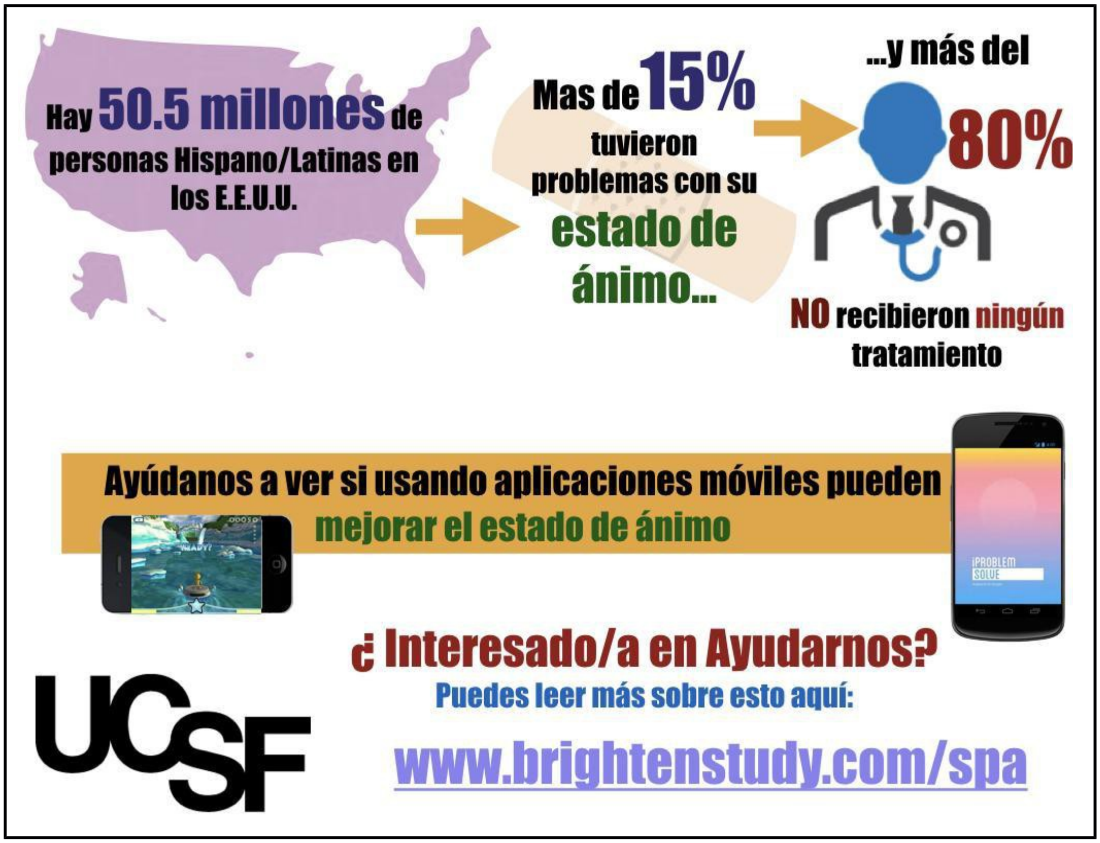

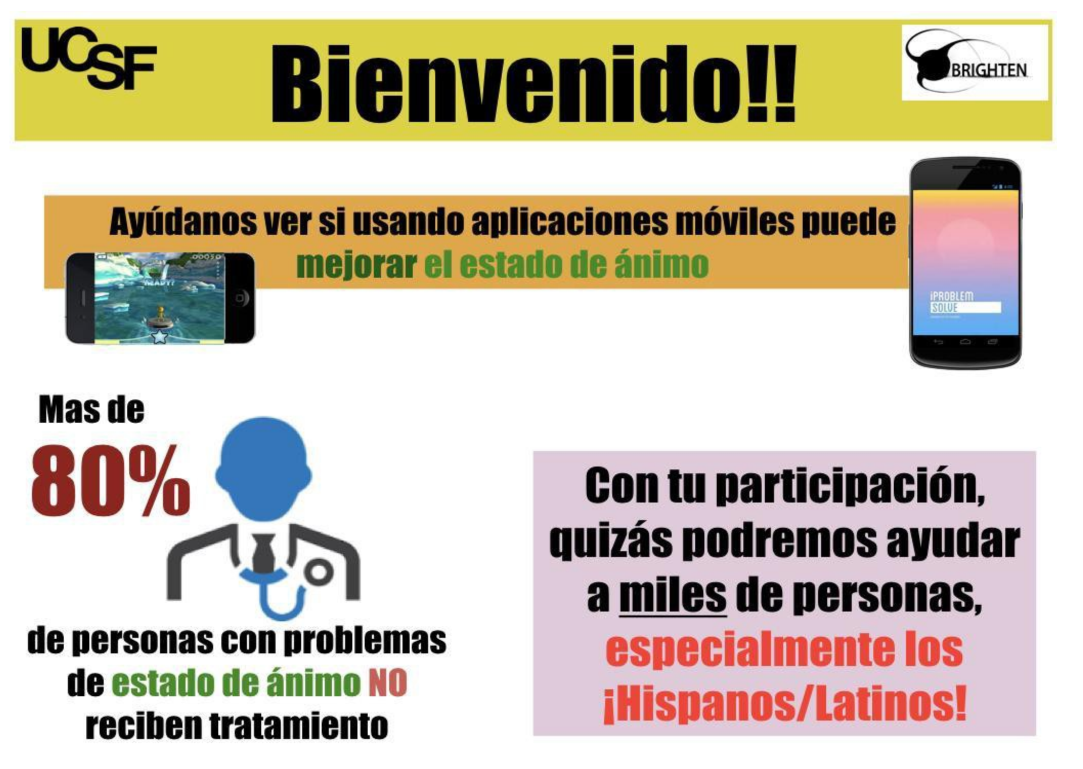

Select Ads used in the study

3) **National Catholic Ministries Hispanic Registry Recruitment:** According to a 2007 Pew Research Center study, 68% of Latinos are Catholic. As mentioned in the parent manuscript, we reached out to Hispanic/Latino Catholic Ministries in at least one city in every state with a custom letter via email describing who we are and our efforts, pointing out that there is no cost (rather, we would provide compensation for those that participate), and provide copies of our advertisements in the hopes that they would be willing to champion this study and post flyers in their communities.

To do this, we used the following website http://www.usccb.org/about/bishops-and-dioceses/all-dioceses.cfm where following a click on each city, one selected the social services link and selected multicultural resources (for example, http://www.bhmdiocese.org/services/multicultural-resources. We also utilized the following wikipedia page to assist with identifying potential locations and partners. https://en.wikipedia.org/wiki/Ecclesiastical_provinces_and_dioceses_of_the_Episcopal_Church

Approaches taken to ensure quality of data

The possibility of individuals looking to take advantage of this study to acquire the research payment was a concern we looked to mitigate in a number of ways. The idea of 'gaming the system' here is not a foregone conclusion, even if the length of time in the study required to receive full payment (12 weeks for $75) would inherently prevent individuals from trying to do this given that the amount of work needed would likely not be all that appealing to most individuals. First, the enrollment web portal would refresh the entire survey if one were to hit the 'back' button on their web browser to change a given answer (see eFigure 3 below).

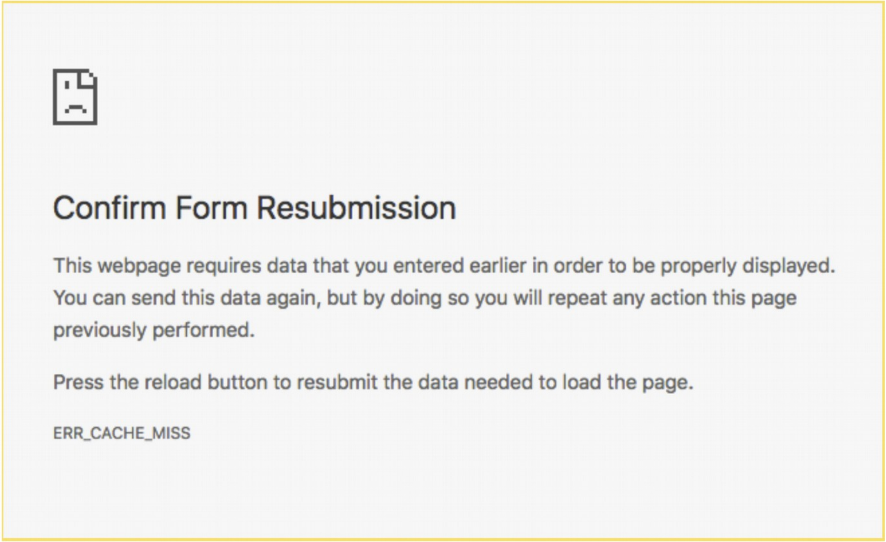

Second, our requirement for both a valid email and phone number (and our ability to monitor if any duplicates of each emerged) restricted the ability to create multiple accounts. If there was anything seemingly amiss with respect to the enrollment questions (e.g. multiple attempted enrollments by an individual using the same email address), we would not enroll the individual, thus preventing them from receiving access to any of the study tools. Finally, the link to download subject specific study tools was only valid for a single user, with a password required to even view the download page. This was to prevent individuals not enrolled in the study or even those interested in the study from seeing what the apps were like (and try to 'game' their way into specific study arms).

Data Acquisition Parameters

Participant data from each application was automatically sent to a secure server via custom API calls in JSON format. This data was used to populate a customized researcher dashboard to provide a quick overview of participant compliance and progress. We used a MongoDB database to capture all data; however, specific summary data extracted from the JSON was also written in parallel to a MySql database for additional summary statistics used to populate participant-specific dashboards to provide an ongoing view of their progress in the study.

Adaptive Cognitive Evaluation for Mental Health (ACEmh)

First described in Anguera et al., (2016; BMJ Innovations) and Arean et al., (2016; JMIR)

During the enrollment period when eligible participants were given a link to download SURVEYTORY, participants were also given a link to download another app to assess their cognitive control abilities associated with attention: ACEmh. ACEmh was a scaled down version of the ACE app used in the aforementioned studies that could be played on an iPhone. ACEmh consisted of only two modules/tasks

(Stroop and an Attention Dot Probe Task) that each utilized adaptive psychometric staircase algorithms to ensure that comparisons between individuals reflect actual differences in that cognitive ability and not disparities in the testing parameters. ACEmh was meant to be used before participants used their study-specific app, and then completed again at the week 4, week 8, and week 12 time points in the study to monitor potential changes in cognitive function. However, only a scant number of participants actually used ACEmh in either group, preventing any meaningful interpretation of any data collected.

Online Spanish Focus Group Survey

Participants who enrolled through the Spanish language portal (www.brightenstudy.org/spa) could access a brief survey asking their impressions of the study This survey was open for five months (10/1/2016-2/28/2017), and was available to Spanish speakers visiting the website or any Spanish-speaking participants who had previously seen our eligibility portal. (See table xxx for details on questions)

553 Spanish-speakers who visited the Spanish enrollment website in V2 also elected to complete the brief online survey about the present study. Of these individuals, 321 participants responded to all questions presented (see Table 4, translated to English here**)**. Responses suggested ample confidence in the researchers, that participation in this type of work was of interest to respondents, and that what was being asked of study participants was reasonable. However, it should be noted that only 67 of the 321 respondents were from the enrolled cohort, and only 16 were from the active cohort; the remaining sample was from individuals who visited the enrollment website but chose to complete the survey rather than attempt the eligibility survey. Thus, impressions listed here may not reflect those of individuals who completed the eligibility survey or actually participated in the trial.

**I believe that...**	**Yes** ***N* (%)**	**No** ***N* (%)**
After seeing the overview video, I would have time to participate in something like this.	309 (96.26)	12(3.47)
I understand what I have to do in this particular study.	307(95.64)	15(4.36)
Beyond money, I am sure that this will help me.	284(88.47)	38(11.53)
I have confidence in these investigators related to this work after seeing the video.	284(88.47)	38(11.53)
I have confidence in this study as it is funded by the NIH.	264(82.24)	60(17.76)
It is sufficient remuneration for the amount of effort needed for participation.	186(57.94)	136(42.06)
I thought and hoped that this study would be something else.	81(25.23)	243(74.77)
There are too many steps (or overly complicated) to participate.	52(16.2)	269 (83.80)
I was annoyed that I was randomized to one of the treatments.	49(15.26)	271(84.74)

Table 4 Summary of responses from the Online Focus Group Findings from Spanish speakers
